# Supplementary material for: Applied phenomics and genomics for improving barley yellow dwarf resistance in winter wheat
Source: G3 (Bethesda). 2022 Mar 30;12(7):jkac064. doi: 10.1093/g3journal/jkac064 (PMC9258586; doi:10.1093/g3journal/jkac064)
Supplement: jkac064_Supplementary_Material [file jkac064_supplementary_material.docx]

## **Supplementary data description**

**Table S1 –** List of wheat entries phenotypically evaluated in the study. The table includes the type of entry (cultivar or breeding line), the season that the entry was evaluated, the result for the prediction of the presence/absence of the segment carrying the resistance gene *Bdv2*, and the best linear unbiased predictors (BLUPs) for all the phenotypic traits collected.

**Table S2 –** Precipitation (inches) during the five field seasons in Riley County, KS, where Rocky Ford and Ashland Bottoms experimental units are located. Normal temperature is defined as a 30–year average from 1981 – 2010. Data was obtained from Kansas State University (<http://climate.k-state.edu/precip/county/>)

Figure S1 – Growth trajectories and adjustment of the non-linear regression model of wheat lines for A-B) normalized difference vegetation index (NDVI) and C-D) digital plant height (meters). The data used correspond to season 2016 – 17 phenotypic data. Calendar days is the number of days starting at January 1, 2017.

Figure S2 – Boxplots showing the phenotypic response of the wheat checks ‘Art’ (susceptible) and ‘Everest’ (tolerant) for A) barley yellow dwarf (BYD) disease severity (%), B) manual plant height (PTHT_M_) (m) and C) grain yield (GY) (tons/ha). Adjusted phenotypic values are shown for both insecticide treatment replications (treated and untreated).

**Figure S3 –** Scatterplots showing distribution and Pearson’s correlation values for the phenotypic traits studied during all the field seasons under two insecticide treatments (treated and untreated). A-B) season 2016 – 17, C-D) season 2017 – 18, E-F) season 2018 – 19, and G-H) season 2019 – 20.

**Figure S4 –** Manhattan plots showing genome-wide association analysis (GWAS) results for the phenotypic traits collected during the study.
